# Role of CD47-SIRPα Checkpoint in Nanomedicine-Based Anti-Cancer Treatment

**DOI:** 10.3389/fbioe.2022.887463

**Published:** 2022-04-26

**Authors:** Haiqin Liao, Chengcheng Niu

**Affiliations:** ^1^ Department of Ultrasound Diagnosis, The Second Xiangya Hospital, Central South University, Changsha, China; ^2^ Research Center of Ultrasonography, The Second Xiangya Hospital, Central South University, Changsha, China

**Keywords:** cd47, SIRPα, tumor, immunotherapy, nanomedicine

## Abstract

Many cancers have evolved various mechanisms to evade immunological surveillance, such as the inhibitory immune checkpoint of the CD47-SIRPα signaling pathway. By targeting this signaling pathway, researchers have developed diverse nanovehicles with different loaded drugs and modifications in anticancer treatment. In this review, we present a brief overview of CD47-SIRPα interaction and nanomedicine. Then, we delve into recent applications of the CD47-SIRPα interaction as a target for nanomedicine-based antitumor treatment and its combination with other targeting pathway drugs and/or therapeutic approaches.

## Introduction

The CD47-SIRPα signaling axis plays an important role in antitumor immunology, tissue homeostasis and remodeling (Logtenberg et al., 2020). Upregulated expression of CD47 on tumor cells increases the interaction with SIRPα on the myeloid cell membrane, leading to a release of the “don’t eat me” signal to evade the phagocytosis of myeloid cells, which is one of the primary mechanisms of cancer and disease formulation (Oldenborg et al., 2000; Jaiswal et al., 2009; Willingham et al., 2012; Hayat et al., 2020; Logtenberg et al., 2020). Hence, increasing studies have focused on the CD47-SIRPα interaction to achieve better therapeutic efficacy for cancer and other diseases (Ho et al., 2015; Petrova et al., 2017; Yanagita et al., 2017). Nevertheless, similar to other conventional medical treatments, the disadvantages of systemic administration of CD47-SIRPα blockade, such as nontargeting distribution, side effects, and short half-life period, have limited its translation to clinical use. These disadvantages can be abated by nanotechnology, which also offers nanotemedicine a promising opportunity to develop. In this review, we will discuss the recent achievements of CD47-SIRPα interaction-based antitumor nanomedicine from the following three aspects: CD47-SIRPα interaction, an overview of nanomedicine, and the role of the CD47-SIRPα checkpoint in nanomedicine-based anticancer treatment.

## Overview of the CD47-SIRPα Checkpoint

### CD47 Structure

The CD47 protein, a member of the membrane protein IG superfamily, is ubiquitously expressed on varieties of types of cellular membranes, especially on senile erythrocytes and cancer cells (Hayat et al., 2020). Its molecular structure includes a single IgV-like extracellular domain at the N terminus, a highly hydrophobic stretch with five membrane-spanning sections and an alternative splicing cytoplasmic domain at its C terminus (Brown and Frazier, 2001; Mushegian, 2002). By interacting with integrin and TSP-1, CD47 is involved in a variety of physiological processes, such as migration, adhesion, proliferation, differentiation (Lindberg et al., 1996; Liu et al., 2001; Lymn et al., 2002). As an inhibitory receptor, CD47 can bind with SIRPα to not only inhibit phagocytosis by phagocytes but also inhibit the activation and maturation of dendritic cells (DCs) (Oldenborg et al., 2000; Latour et al., 2001; Lutz and Bogdanova, 2013). In addition, CD47-SIRPα also regulates neuron development and bone remodeling (Barclay, 2009; Maile et al., 2011).

### SIRPα Structure

SIRPα, a member of the Ig superfamily (IgSF), consists of three Ig-like extracellular domains at its N terminus four tyrosine phosphorylation sites and two immunoreceptor tyrosine inhibitory motifs (ITIMs) in its cytoplasmic domain (Adams et al., 1998; Hayat et al., 2020). In contrast to the ubiquitous expression of CD47, SIRPα is limitedly expressed on macrophages, monocytes, granulocytes and neurons (Adams et al., 1998).

### CD47-SIRPα Interaction

**FIGURE 1:**
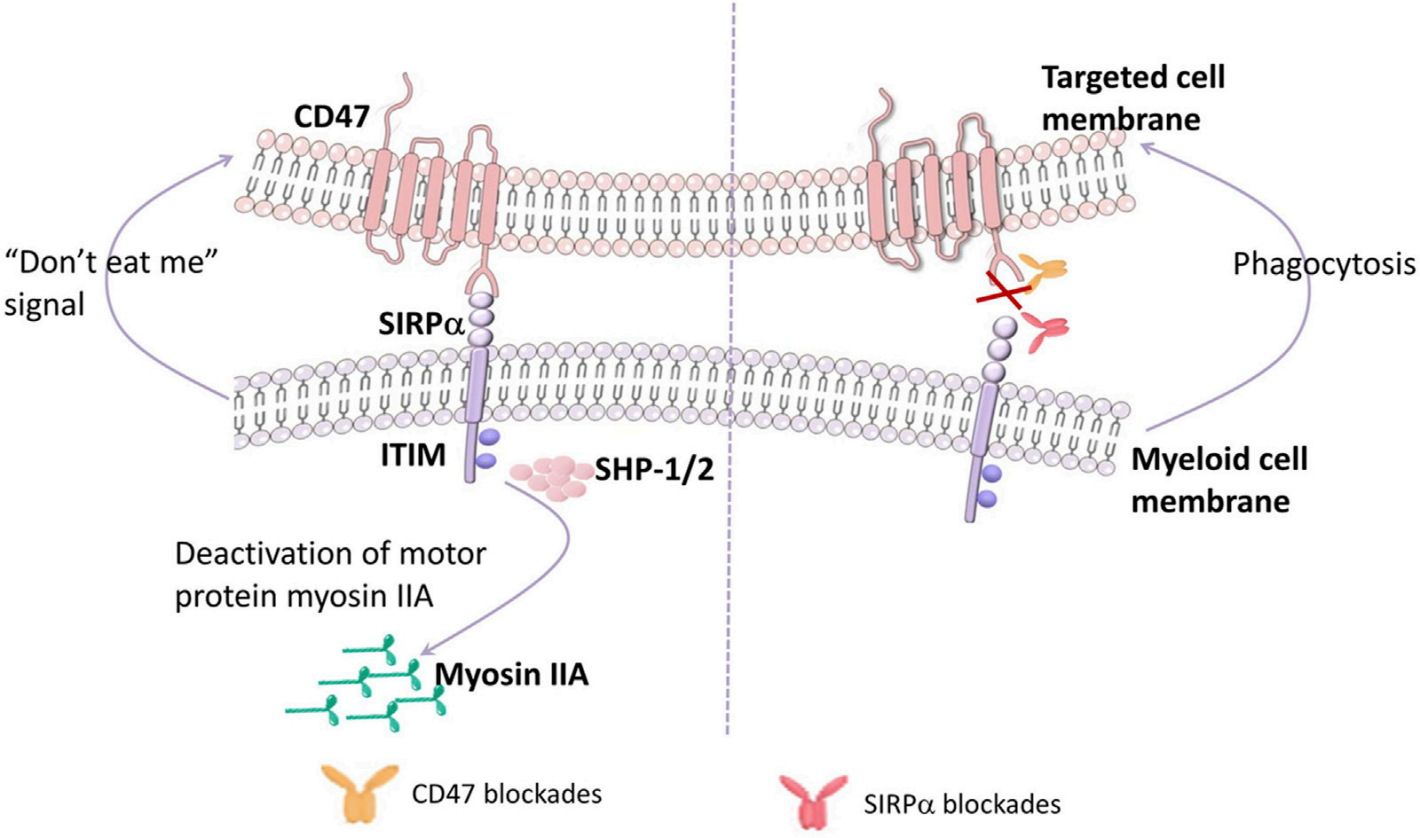
CD47 interacts with SIRPα. The N-terminal Ig-like extracellular domain of SIRPα on myeloid cells binding to the N-terminal IgV-like extracellular domain of CD47 on targeted cells induces the phosphorylation of ITIM of SIRPα, then recruits and activates the protein tyrosine phosphatases, especially Src homology 2 (including SHP-1 and SHP-2). Upon activated SHP-1/2 engagement, myosin IIA dephosphorylation Occurs, releasing the “don’t eat me” signal, leading to an inhibition of phagocytosis. On the contrary, inhibiting the CD47-SIRPα pathway activates the phagocytosis by myeloid cells.

The N-terminal Ig-like extracellular domain of SIRPα binds with the N-terminal IgV-like extracellular domain of CD47, resulting in the phosphorylation of ITIM of SIRPα and recruitment and activation of protein tyrosine phosphatases, especially Src homology 2 including SHP-1 and SHP-2, dephosphorylation of the downstream molecule ITAM, the accumulation of myosin IIA damage in the phagocytic synapse, releasing the “don’t eat me” signal, leading to an inhibition of phagocytosis (Hayat et al., 2020; Logtenberg et al., 2020; Figure 1).

Homeostasis of cells and tissues depends on a balance of regulation of pro-phagocytic signals [calreticulin (CRT)-low density lipoprotein-receptor related protein-1 (LRP-1), Fcγ, complement receptor] and anti-phagocytic signals (CD47-SIRPα) (Oldenborg et al., 2001; Chao et al., 2010a). Chao et al. demonstrated that CRT plays a leading role in pro-phagocytic signals and is essential for anti-CD47 antibody therapy in multiple human cancers. In their study, the *in vitro* phagocytosis assays were performed by incubating primary human normal cells and cancer cells with human macrophages with a therapeutic dosage of anti-CD47 antibody, and showed that primary cancer cells were obviously phagocytized, whereas no phagocytosis of normal cells was observed, suggesting that blocking the CD47-SIRPα is not the only rationale for pro-phagocytosis (Chao et al., 2010a). Note that the anti-CD47 antibody with intact Fcγ should be utilized with caution given the pro-phagocytosis role of Fcγ, which could increase systemic toxicity by enhancing antigen sink effects (Ingram et al., 2017).

Increasing studies have concluded that the antitumor effect mediated by blocking the CD47-SIRPα interaction mainly owes to the activation of innate immune responses [including phagocytosis by macrophages and the antibody dependent cellular cytotoxicity (ADCC) by neutrophil granulocytes] (Jaiswal et al., 2009). However, it is important to note that the results of these works were based on xenograft models, which may favor innate immune responses to kill tumor cells with some unique features (Majeti et al., 2009; Chao et al., 2010b; Willingham et al., 2012). Liu et al. used syngeneic immune-competent mouse models to exclude these effects (Liu et al., 2015a). In this study, the mouse anti-CD47 antibody showed an evident antitumor effect, especially by intratumoral delivery, and the therapeutic effect was diminished when CD8^+^ T cells were depleted. In addition, CD47-SIRPα blockade activates the maturation of DCs and boosts DC-mediated antigen cross-presentation and cytotoxic T lymphocyte induction. Hence, the CD47-SIRPα signaling axis is an inhibitory checkpoint that bridges innate and adaptive immunity for tumor evasion.

### Strategies for Inhibiting the CD47-SIRPα Interaction

According to the different signaling pathway blocking sites, the strategies for inhibiting the CD47-SIRPα interaction can be divided into three types: molecules that inhibit the CD47 protein on the tumor cells, molecules that inhibit SIRPα protein on the myeloid cells, and inhibitors of the glutaminyl-peptide cyclotransferase-like (QPCTL) enzyme, which is necessary for the maturation of CD47 protein (Logtenberg et al., 2020).

The strategy targeting CD47 on tumor cells has been the most commonly studied. For instance, Hu5F9-G4, a humanized anti-CD47 antibody with a human immunoglobulin G4, has been proved a potent antitumor effect in preclinical experiments and clinical trials. In a malignant pediatric brain tumor-bearing mouse model, administration of Hu5F9 evidently inhibited tumor growth and showed significant survival benefit (Gholamin et al., 2017). However, CD47 is not only over-expressed on cancer cells, but also expressed on normal cells, such as ethrocytes. Therefore, administration of CD47 blocking agents would lead to anemia and “antigen sink” effect. In the development of CD47 targeting agents, multiple approaches have been employed to solve these problems, such as change in the mode of administration (Liu et al., 2015b; Advani et al., 2018; Sikic et al., 2019); dual targeting bispecific antibodies of CD47 (Dheilly et al., 2017; Shi et al., 2020; Wang et al., 2021); CD47 antibodies/SIRPα fusion proteins (Petrova et al., 2017; Meng et al., 2019; Puro et al., 2020; Andrejeva et al., 2021). From the efficacy of view, dual targeting bispecific antibodies are more promising. Wang et al. designed a CD47-PD-L1 bi-specific antibody, named IB322 (Wang et al., 2021). As a dual inhibitor of innate and adaptive immune checkpoint, IB322 efficiently triggered the tumor cell phagocytosis by macrophages and killing effect by T cells and induced complete tumor regression *in vivo*. Moreover, IB322 showed negligible RBCs depletion and was well tolerated in non-human primates.

Compared with the wide expression of CD47, SIRPα is restrictedly expressed on myeloid cells and neurons (Adams et al., 1998). Hence, biologicals that target SIRPα do not suffer from amenia and “antigen sink” issues (Yanagita et al., 2017; Voets et al., 2019). For example, Ho et al. developed an engineered, high-affinity, CD47 variant (termed Vecro-CD47), which could remarkably increase the affinity to wild-type (WT) SIRPα and disrupt the CD47-SIRPα interaction, thereby promoting macrophage phagocytosis of tumor cells (Ho et al., 2015). Voets et al. developed a humanized mAb ADU-1805, which inhibits the CD47-SIRPα signaling pathway by closely binding with SIRPα, showing similar antitumor efficacy as the anti-CD47 antibody with good safety *in vitro* and *in vivo* (Voets et al., 2019). However, researches and clinical trials focused on SIRPα blocking target are fewer than that on CD47 blocking target.

Inhibitors of QPCTL enzyme is another promising strategy that does not result in anemia easily and “antigen sink” issues (Ingram et al., 2017). Logtenberg et al. reported that both genetically and pharmacologically blocking QPCTL activity enhanced antibody-dependent cellular phagocytosis (ADCP) and ADCC of tumor cells (Logtenberg et al., 2019). Moreover, the intervention of QPCTL activity can alter the immunosuppressive tumor microenvironment (monocyte skewed, myCAF, TGF-β) to a proinflammatory (macrophage skewed, iCAF, IFN) milieu, and enhances the therapeutic effect of anti-PD-L1 therapy (Bresser et al., 2022).

To date, over 20 CD47/SIRPα blocking agents have been employed in clinical trials (summarized in Table 1), involving in both hematological malignancies and solid tumors. However, the current clinical trial data of QPCTL inhibitors in antitumor treatment are lacking.

**TABLE 1:** List of anti-tumor clinical trials targeting CD47-SIRPα axis.

No	Drug	Target	Composition	Fc type	Phase	NCT No	Condition or disease	status
1	HX009	CD47*PD-1	Recombinant humanized bi-functional Ab	Unknown	Phase I/II	NCT05189093	Relapsed/refractory lymphoma	Recruiting
					Phase I/II	NCT04886271	Advanced solid tumor	Recruiting
					Phase I	NCT04097769	Advanced malignancies	Active, not recruiting
2	Hu5F9-G4	CD47	Humanized mAb	IgG4	Phase I	NCT05169944	Recurrent or progressive malignant brain tumors	Not yet recruiting
					Phase I	NCT03248479	Hematological malignancies	Active, not recruiting
					Phase II	NCT04788043	Relapsed or refractory classic hodgkin lymphoma	Not yet recruiting
					Phase I	NCT03527147	Relapsed/refractory aggressive NHL	Completed
					Phase I	NCT02216409	Solid tumor	Completed
					Phase I	NCT02678338	AML	Completed
3	AK117	CD47	Humanized mAb	IgG4	Phase I/II	NCT04900350	Myelodysplastic syndrome	Recruiting
					Phase Ib/II	NCT05214482	Advanced malignant tumors	Recruiting
					Phase I	NCT04728334	Neoplasms malignant	Recruiting
					Phase I	NCT04349969	Neoplasms malignant	Not yet recruiting
					Phase Ib/II	NCT05229497	Advanced malignant tumors	Not yet recruiting
					Phase Ib/II	NCT05235542	Advanced malignant tumors	Not yet recruiting
4	IBI188	CD47	mAb	IgG4	Phase I	NCT03717103	Advanced malignancies	Active, not recruiting
					Phase I	NCT03763149	Advanced Malignancies	Completed
5	Gentulizumab	CD47	mAb	Unknown	Phase I	NCT05221385	Solid tumor/NHL	Recruiting
					Phase I	NCT05263271	AML/myelodysplastic syndromes	Recruiting
6	STI-6643	CD47	Humanized mAb	IgG4	Phase I	NCT04900519	Advanced solid tumors	Recruiting
	PF-07257876	CD47*PD-L1	Bispecific ab	Unknown	Phase I	NCT04881045	NSCLC/HNSCC/ovarian cancer	Recruiting
7	TTI-621	CD47	Humanized SIRPα-Fc fusion protein	IgG1	Phase I	NCT02663518	Hematologic malignancies/Solid tumor	Recruiting
					Phase I	NCT05139225	Multiple myeloma	Recruiting
					Phase I/II	NCT04996004	Leiomyosarcoma	Recruiting
8	TTI-622	CD47	Humanized SIRPα-Fc fusion protein	IgG4	Phase I/II	NCT05261490	Platinum-resistant ovarian cancer	Recruiting
					Phase I	NCT03530683	Advanced hematologic malignancies	Recruiting
9	TQB2928	CD47	mAb	Unknown	Phase I	NCT05192512	Advanced cancer	Recruiting
					Phase I	NCT04854681	Advanced solid tumors/hematological malignancies	Not yet recruiting
10	SG2501	CD47*CD38	Bispecific ab	Unknown	Phase I	NCT05293912	Hematological malignancy Lymphoma	Not yet recruiting
11	AO-176	CD47	Humanized mAb	IgG2	Phase I/II	NCT03834948	Multiple solid tumor malignancies	Recruiting
					Phase I/II	NCT04445701	Relapsed/refractory multiple myeloma	Recruiting
12	IMC-002	CD47	Humanized mAb	IgG4	Phase I	NCT05276310	Advanced cancer	Not yet recruiting
					Phase I	NCT04306224	Solid tumor/lymphoma	Recruiting
13	CPO107	CD47*CD20	Bispecific SIRPα fusion protein	Unknown	Phase I/II	NCT04853329	CD20 positive NHL	Recruiting
14	ALX148	CD47	Fusion protein	Human inert IgG1γ	Phase I/II	NCT05025800	Indolent and aggressive B-cell NHL	Recruiting
					Phase I/II	NCT04417517	Higher risk myelodysplastic syndromes	Recruiting
					Phase I	NCT03013218	Advanced solid tumors/lymphoma	Active, not recruiting
					Phase II	NCT04675333	Advanced HNSCC	Recruiting
					Phase I/II	NCT04755244	AML	Recruiting
					Phase II/III	NCT05002127	Advanced HER2+ gastric cancer	Recruiting
					Phase II	NCT04675294	Advanced HNSCC	Recruiting
					Phase II	NCT05167409	Microsatellite stable metastatic colorectal cancer	Not yet recruiting
15	IBI322	CD47*PD-L1	Bispecific ab	Unknown	Phase I	NCT04795128	Hematologic malignancy	Recruiting
					Phase I	NCT04338659	Advanced malignancies	Not yet recruiting
					Phase I	NCT04912466	Advanced solid tumor	Not yet recruiting
					Phase I	NCT05148442	Myeloid tumor	Not yet recruiting
					Phase I	NCT04328831	Advanced malignancies	Recruiting
16	IMM2902	HER-2*CD47	Humanized bispecific mAb	IgG1	Phase I	NCT05076591	Advanced solid tumors	Not yet recruiting
17	BAT7104	CD47*PD-L1	Bispecific ab	IgG	Phase I	NCT05200013	Advanced solid tumors	Not yet recruiting
18	IBC0966	CD47	Unknown	Unknown	Phase I/IIa	NCT04980690	Advanced malignant tumors	Not yet recruiting
19	TG-1801	CD47*CD19	Bispecific ab	Unknown	Phase I	NCT04806035	B-cell lymphoma/CLL	Recruiting
20	SL-172154	CD47*CD40	Fusion protein consisting of human SIRPα and CD40L	Unknown	Phase I	NCT04502888	HNSCC	Active, not recruiting
					Phase I	NCT04406623	Ovarian Cancer/	Recruiting
					Phase I	NCT05275439	AML/myelodysplastic syndrome	Not yet recruiting
					Phase I	NCT04502888	HNSCC	Not yet
21	BI 765063	SIRPα	mAb	Unknown	Phase I	NCT03990233	Advanced solid tumors	Recruiting
					Phase I	NCT05249426	HNSCC	Recruiting
22	CC-95251	SIRPα	mAb	Unknown	Phase I	NCT03783403	Advanced solid and hematologic cancers	Recruiting
23	DSP107	SIRPα*4-1BBα	Bi-functional, trimeric, fusion protein	Unknown	Phase I/II	NCT04440735	Advanced solid tumor/NSCLC	Recruiting
					Phase I	NCT04937166	Hematological malignancies	Recruiting
24	GS-0189	SIRPα	Unknown	Unknown	Phase I	NCT04502706	NHL	Recruiting
25	SRF231	CD47	Humanized IgG4 mAb	Unknown	Phase I/Ib	NCT03512340	Advanced solid cancers/Hematologic cancers	Completed

ab antibody; mAb monoantibody; NHL, Non-Hodgkin lymphoma; AML, acute myelogenous leukemia; NSCLC, non-small cell lung cancer; HNSCC, squamous cell carcinoma of the head and neck; CLL, chronic lymphocytic leukemia.

All data were collected from https//www.clinical trials.gov/ on 27 Mar 2022.

Although promising, the CD47-SIRPα blocking agents still face some challenges that restrict their translation to clinical settings. For instance, the ubiquitous expression of CD47 indicates that large dose or frequent administration of anti-CD47 antibodies is required (eg: antigen sink effect) (Chen et al., 2022), suggesting the efficacy of anti-CD47 antibodies treatment is relatively low. With regard to targeting bispecific antibodies of CD47 and CD47 antibodies/SIRPα fusion protein technology, while promising, it requires complex design and isolation (Chen et al., 2013; Labrijn et al., 2019). Therefore, the cost of these therapies is usually unaffordable for patients, which limits their clinical applications (Chen et al., 2013; Labrijn et al., 2019). As for SIRPα targeting strategy, it constantly fail to induce ADCP and ADCC against cancer cells when administrated alone due to immune cells target (Chen et al., 2013; Ring et al., 2017; Labrijn et al., 2019; Chen et al., 2022). The demand for safer and more efficient drug delivery is therefore increasing.

Nanomedicine, defined as the application of nanotechnology, can meet this need. Nanotechnology enables therapeutic drugs to target sites with high spatial and temporal resolution, prolonged half-life and great convenience for combination therapy. Therefore, CD47-SIRPα targeting based nanomedicine holds great potential in antitumor field, which will be reviewed in more detail in the following sections.

## Overview of Nanomedicine

The efficacy of drugs has been limited, due to nonspecific distribution, side effects and short circulation time, offering an evolutionary opportunity for nanomedicine to circumvent these drawbacks and improve therapeutic efficacy. Diverse applications of nanomedicine have been investigated in multiple areas, such as drug delivery, vaccine development, diagnosis, and imaging tools (Pelaz et al., 2017). In this section, we mainly focus on the application of nanomedicine in drug delivery.

### Type of Nanoparticles

Nanoparticles (NPs) are important components of nanomedicine. The unique characteristics of NPs, such as large surface-volume ratio, small size, capacity to encapsulate various drugs, and tunable surface chemistry, provides themselves a large variety of advantages, including multivalent surface modification, efficient navigation *in vivo*, increased intracellular trafficking and sustained release of drug payloads (Xu et al., 2015). Currently, diverse types of NPs exist, including liposomes (Huynh et al., 2009; Wang et al., 2016; Olusanya et al., 2018; Yang et al., 2021), micelles (Torchilin, 2007; Tawfik et al., 2020), poly (lactic-co-glycolic acid) (PLGA) (Sadat Tabatabaei Mirakabad et al., 2014; Rezvantalab et al., 2018), graphene (Diez-Pascual, 2020), graphene oxide (Kinnear et al., 2017; Diez-Pascual, 2020), protein nanoparticles (Lohcharoenkal et al., 2014; Jain et al., 2018), extracellular vesicles (EVs) (S et al., 2013; Si et al., 2022; Logozzi et al., 2021), exosomes (De La Peña et al., 2009; Nie et al., 2020; Xia et al., 2020; Logozzi et al., 2021), magnetic NPs (MNPs) (Colombo et al., 2012; Wu et al., 2019; Farzin et al., 2020), mesoporous silica NPs (MSNPs) (Fu et al., 2013; Wang et al., 2015; Pelaz et al., 2017; Rastegari et al., 2021), and metal-organ frameworks (MOFs) (Zheng et al., 2016; Wu and Yang, 2017; Xing et al., 2020), Ferritin (Lee et al., 2017; Cho et al., 2018; Sun et al., 2021). Detailed information about the charaterizations, advantages and disadvantages of each type of NP is summarized in Table 2. As summarized in Table 2, while each nanocarrier possesses unique merits, they still face certain problems that restrict their optimal performance in the drug delivery system.

**TABLE 2:** Detailed information about the charaterizations, advantages and disadvantages of each type of nanomaterials.

Types of NPs	Characterizations	Advantages	Disadvantages	References
Lipid NPs	Phospholipid molecules which contain hydrophobic tails and hydrophilic heads, forming the amphiphilic vesicle structures in aqueous solutions	Entrapment of both hydrophilic and hydrophobic compounds, high loading capacity, convenient preparation, excellent biocompatibility	Structural instability	(Huynh et al., 2009; Wang et al., 2016; Olusanya et al., 2018; Yang et al., 2021)
Micelles	Self-assembling nanosized colloidal particles with a hydrophobic core and hydrophilic shell	High-efficiency lipophilic drug entrapment, high stability and good biocompatibility	Inability to encapsulate poorly soluble drugs and toxicity	(Torchilin, 2007; Tawfik et al., 2020)
PLGA	A catalyzed ring-opening copolymerization of glycolic acid and lactic acid	High loading capacity, convenient preparation and excellent biocompatibility, minimal systemic toxicity	Poor drug loading capacity, high burst release of drug from nanoparticles, the production of acids upon degradation	(Sadat Tabatabaei Mirakabad et al., 2014; Rezvantalab et al., 2018)
Graphene	A single monolayer of graphite	Exceptional thermal, mechanical, and electronic properties.	Poor solubility	Diez-Pascual, (2020)
Graphene oxide	Oxidized form of graphene that contains epoxides, hydroxyls, and carbonyls on the basal planes and carboxyls on the edges	Highly hydrophilic, aqueous processability, amphiphilicity, surface functionalization capability, and versatility	Low thermal conductivity, electrically insulation	(Kinnear et al., 2017; Diez-Pascual, 2020)
Protein nanoparticles	Natural molecules that have unique functionalities and potential applications in both biomedicaland material sciences	Non-toxicity, weak immune response, easy metabolizability, excellent scope of surface modification, good biocompatibility and biodegradability	Variable size range, immunogenicity, structural change leading to change of the original property of native protein, biphasic drug release pattern with initial burst release.	(Lohcharoenkal et al., 2014; Jain et al., 2018)
EVs	40 nm to a few μM sized lipid bilayer membrane vesicles	Innate homing capacity, low immunogenicity and enhanced circulation retention	Low production yield, insufficient encapsulating of cargos	(S et al., 2013; Si et al., 2022; Logozzi et al., 2021)
Exosomes	70–150 nm sized lipid bilayer membrane vesicles	Innate homing capacity, good biocompatibility, near non-immunogenicity, long-circulation and non-toxic	Low production yield, insufficient encapsulating of cargos	(De La Peña et al., 2009; Nie et al., 2020; Xia et al., 2020; Logozzi et al., 2021)
MNPs	Fabricated from pure metals (Fe, Co., Ni, and some rare earth metals) or a mixture of metals and polymers	Superparamagnetism, magnetic navigation ability, increasing imaging resolution in image methods, high chemical and colloidal stability, and low cost	Relatively low biocompatibility, insufficient magnetic strength, low drug loading capacity, and difficulty in tuning their size	(Colombo et al., 2012; Wu et al., 2019; Farzin et al., 2020)
MSNPs	Porous solid materials with inorganic siloxane structures	Selective surface functionality, high loading capacity, controlled morphology and release properties, ability to encapsulate poorly soluble drug and co-deliver different drugs and good biocompatibility	Relatively low biodegradability, inflammatory response around the injection sites after intramuscular and hypodermic injection	(Fu et al., 2013; Wang et al., 2015; Pelaz et al., 2017; Rastegari et al., 2021)
MOFs	Porous coordination polymer which is composed of metals or metal clusters, chains or layers formed by non-toxic metals (Fe, Zn, Ca, Mg, etc.) and organic compounds, such as carboxylic acid and phosphonic acid	Well-defined pore aperture, tailorable composition and structure, tunable size, versatile functionality, high agent loading, and improved biocompatibility	Relatively low stability and biodegradability	(Zheng et al., 2016; Wu and Yang, 2017; Xing et al., 2020)
Ferritin nanocages	spherical hollow nanocage that can bind approximately 4500 iron atoms	chemically and genetically modifiable ferritins external surface, natural tumor targeting ability, strong loading capacity, good stability	Inability to display ligands containing transmembrane domains, steric hindrance between ligand constraining the types of ligands	(Lee et al., 2017; Cho et al., 2018; Sun et al., 2021)

NPs, nanoparticles; PLGA, poly (lactic-co-glycolic acid); EVs, extracellular vesicles; MNPs, magnetic nanoparticles; MSNPs, mesoporous silica nanoparticles; MOFs, metal-organ framework.

### Targeted Delivery Capacity of NPs

Targeted delivery capacity, including passive targeting and active targeting, is a key feature of nanomedicine and has been vastly studied.

Passive targeting is achieved by the enhanced permeability and retention (EPR) effect, prolonging the half-life of NPs in the body circulation (Maeda et al., 1999; Fang et al., 2003). Thus, prolonging the circulation time of NPs is a good strategy to increase their accumulation at the tumor site. The stealth modification of NPs is of great importance. Camouflaging the surface with polyethylene glycol (PEG) is the most common way to create a protective layer for encapsulated cargo by reducing the absorption of plasma proteins and extending the half-life of NPs (Gref et al., 2000). In addition to PEGylation, new biomaterials and drug delivery strategies have been developed to prolong the circulation time of NPs, including zwitterionic polymer-coating (Zhu et al., 2014), minimal self-peptides (e.g., CD47-derived self-peptides) (Rodriguez et al., 2013), and biomimetic membrane-coating (Hu et al., 2011). Nevertheless, passive targeting is far to reach the requirement of therapeutic efficacy. Due to the phagocytosis of mononuclear phagocytes, the majority (more than 90%) of NPs are inevitably entrapped by reticuloendithelial organs, such as liver and spleen (Albanese et al., 2012).

To overcome this severe drawback, measures that make nanomedicine actively target the disease site should be taken to increase the accumulation of drugs at the target site and subsequently enhance the therapeutic efficacy. Active targeting is a strategy to achieve the goal of orientation in space and simultaneously eliminates the off-target effect in normal tissues by intentionally guiding NPs to the disease site. A common approach to active targeting is to decorate the appropriate ligands to the surface of NPs. These ligands interact with the surface receptor of target cells inducing receptor-mediated endocytosis (Chen et al., 2017). Target agents can be broadly categorized as proteins (mainly antibodies and their fragments), nucleic acids (aptamers), or other receptor ligands (Peer et al., 2007).

### Controlled Drug Release

To release the drug at a specific site and time, various efforts have been made to develop stimuli-responsive NPs, which further enhance the therapeutic efficacy (Li et al., 2020). These stimuli-responsive NPs can be stimulated by either endogenous stimuli-responsive strategies, such as pH variation, redox, enzyme, hypoxia, or exogenous stimuli-responsive strategies, such as light, ultrasound, magnetic field, temperature (Tawfik et al., 2020)*.* However, these single stimuli-responsive strategies still face some challenges. For example, several temperatue- and light-responsive agents can damage normal cells and even tissues and organs (Wu et al., 2018). Due to insufficient H_2_O_2_ levels in tumor tissues, the nonspecificity and low therapeutic efficiency of H_2_O_2_-responsive nanoplatforms are also key challenges for clinical translation (Chang et al., 2017). Nanoassemblies activated by both exogenous stimuli and endogenous stimuli have gained tremendous attention by virtue of the enhanced encapsulated payload and the higher accuracy of spatiotemporal release. For instance, chen et al. developed a photothermal-PH-hypoxia responsive multifunctional nanoplatform (TENAB NP) for cancer photochemotherapy, for synergistic chemo-phototherapy with minimized skin photosensitization (Chen et al., 2019a). In this multistimuli responsive drug delivery system, tirapazamine, the hypoxia-specific prodrug, and ENAB, the pH-responsive photosensitizer, were encapsulated into the phase change materials (LASA), a mixture of linoleic acid and stearyl alcohol.

## Role of the CD47-SIRPα Checkpoint in Nanomedicine-Based Disease Treatment

As previously described, systemic administration of CD47-SIRPα blocking agents has led to remarkable achievements, but the concomitant side effects (e.g., anemia) and limitations have restricted their translation to clinical use. To address these issues, nanotechnology has been introduced to reduce the side effects and enhance the stability and efficacy of the drug and the possibility of controlled release. In this section, we review the recent advances in the role of the CD47-SIRPα checkpoint in nanomedicine-based disease treatment.

### Blocking the “Don’t Eat Me” Signal of the CD47-SIRPα Interaction

**FIGURE 2:**
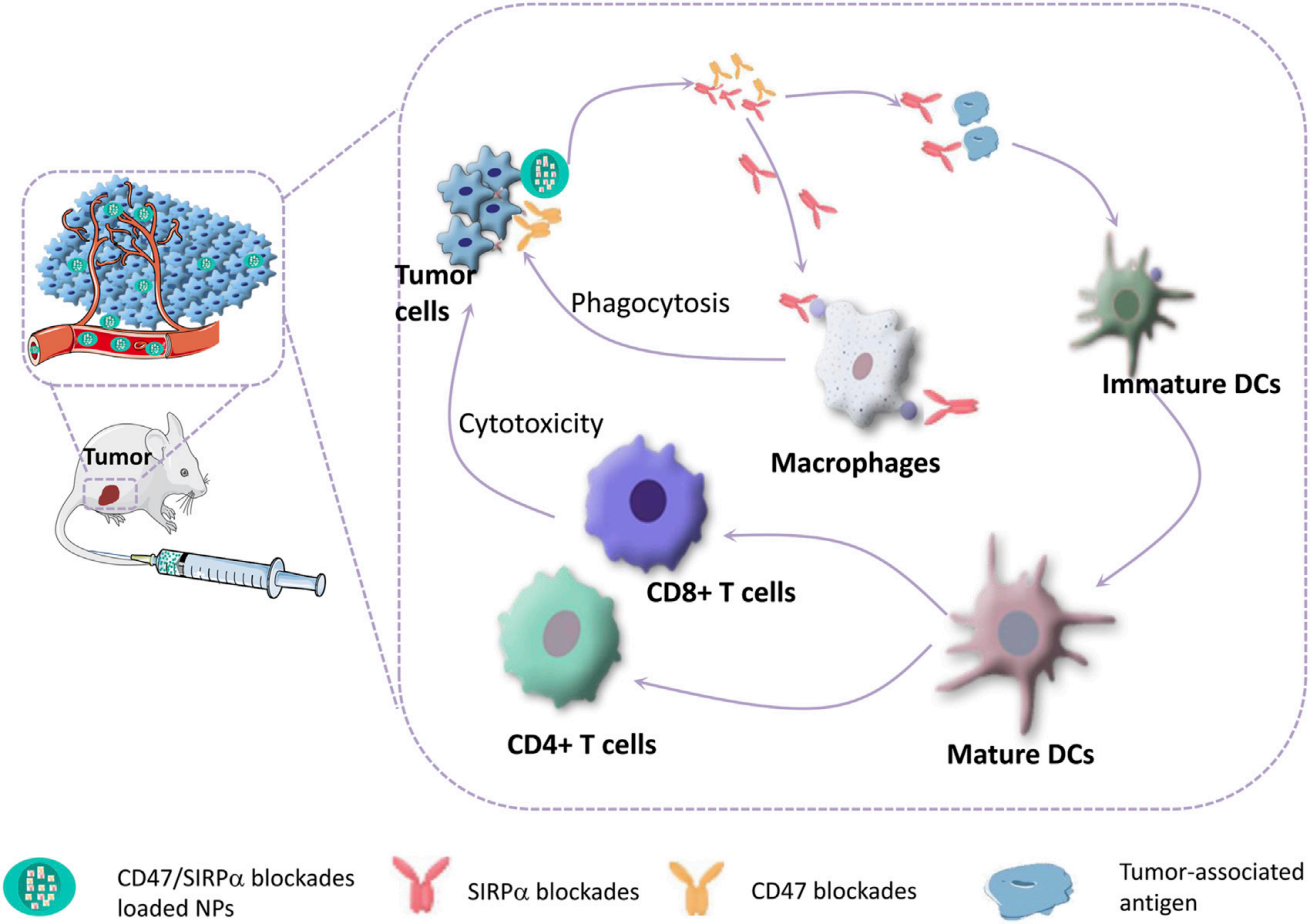
CD47-SIRPα blockades can bridge innate and adaptive antitumor immunity. Blocking of CD47-SIRPα signaling axis can activate macrophages to phagocytize tumor cells, and promote the maturation of DCs, which enhances DC-mediated tumor-associated antigen presentation, thereby triggering T-cell mediated destruction of tumor cells.

The upregulated CD47 on tumor cells increases its interaction with SIRPα on macrophages, resulting in an evasion of immunological surveillance and the muturation of DCs (Willingham et al., 2012; Jaiswal et al., 2009; Liu et al., 2015a; Liu et al., 2017). This inhibitory checkpoint paves the way for therapeutic strategies involving blocking this interaction to enhance innate and adaptive immunity for tumor killing (Figure 2). For instance, Koh et al. constructed an exosome through surface engineering with SIRPα variants termed SIRPα-exosomes, which can bind to both human and mouse CD47 as antagonists. The therapeutic efficacy of these engineered exosomes was verified in HT27 tumor-bearing mice (including immunodeficient and immunocompetent mice). Systemic administration of SIRPα-exosomes induced significant regression of tumor growth in immunocompetent mice, while tumor growth was slightly reduced in immunodeficient mice, suggesting that T-cell immunity might be essential to maximize the antitumor effect of CD47 blockade therapy (Koh et al., 2017). Ramesh et al. developed a multivalent lipid-based phagocytosis nanoenhancer with the conjugation of anti-CD47 and anti-SIRPα antibodies (LPN). LPN treatment showed remarkable tumor growth suppression and increased survival in B16F10 tumor-bearing mice with no systemic toxicity (Ramesh et al., 2020).

However, cancers are very complex diseases involving multiple pathways, and their progression is associated with various continuous mutations in cell lines. In addition, in order to survive, tumor cells mutate as chemotherapy progresses, thereby resulting in intrinsic and acquired resistance to chemotherapeutics (Iyer et al., 2013). Hence, the inhibition of the CD47-SIRPα signaling pathway is not sufficient to fight against tumor, and often requires a combination of blockades of different pathways, genes or chemotherapeutics (Xu et al., 2015). The advantages of nanomedicine, such as the capacity of encapsulating different drugs, targeted delivery and controlled release, offer a great opportunity for combination therapy for tumors. Some examples to show how the combination of blockade of the CD47-SIRPα interaction and other therapeutics or modalities works in nanomedicine are listed in Table 3 and explained in the next section.

**TABLE 3:** Examples of nanoparticle-based combination therapy utilized blocking CD47-SIRPα signal axis.

	Platform type	Responsive release modality	modification modality	encapsulated drug or combination drug	Targets	Tumor model	Administration route	Results	Ref.
Combined with reprograming the TAMs	liposome	MMP2-responsive	PEG coating, conjugation of aCD47	PTX	CD47-SIRPα	MDA-MB-231 tumor- bearing and tumor metastasis mice	intravenous (i.v.)	Inhibited tumor growth and metastasis	Chen et al. (2021)
	M1 derived exosome	pH-responsive benzoic-imine bond.	Azide, conjugation of DBCO modified aSIRPα and aCD47		CD47-SIRPα	4T1 tumor-bearing mice	i.v.	Enhanced the phagocytosis of macrophages via blocking the “don’t eat me” signaling, resulting in potent anticancer efficacy with minor side effects	Nie et al. (2020)
	MNPs	Magnetic-responsive	gCM coating	–	CD47-SIRPα	B16F10 tumor-bearing mice, 4T1 tumor metastasis model	i.v.	Prolonged overall survival by controlling both local growth and distant metastasis	Rao et al. (2020a)
	Hybrid NVs fused by M1-NVs/Plt-NVs and cancer cell-NVs gene engineered with SIRPα variants	–	–	–/cGAMP	CD47-SIRPα/CD47-SIRPα, STING pathway	B16F10 incomplete-tumor resection mice/post-surgery 4T1 tumor-bearing mice	i.v.	Reduced tumor recurrence and lung metastasis, improved the survival rate, effectively controlled the tumor recurrence and inhibited lung metastasis	Rao et al. (2020b)
	hierarchical gel matrix and graphene oxide	NIR-responsive	–	Sorafenib, aCD47	CD47-SIRPα	post-surgery 4T1 tumor-bearing mice	intratumoral(i.t.)	Prevented tumor recurrence and metastasis by locally reversing the immunosuppression and synergistically blocking the CD47-dependent immune escape, thereby boosting the systemic immune responses	Ramesh et al. (2019)
	Liposomes	Esterase-responsive	PEG coating	BLZ945, SHP099	CD47-SIRPα, MCSF-CSF1R	B16F10 tumor-bearing mice, 4T1 tumor metastasis bearing mice	i.v.	Reversed the immunosuppression and inhibited the tumor growth	Huang et al. (2021)
	ZIF-8-based nanocages	pH-responsive	MnO_2_, aCD47 conjugation	siIDO-1, GE	CD47-SIRPα, IDO-1	CT26 tumor-bearing mice	i.v.	Inhibited the tumor growth and prolonged the survival	Chen et al. (2020)
Combined with chemotherapy	Caspase-cleavable peptide-DOX conjugate + SIRPα- expressing ferritin nanocages	Radiation-induced release of caspase-3	–	–	CD47-SIRPα	CT-26-tumor-bearing mice	i.v.	Resulted in tumor eradication in 8 out of 9 mice	Lee et al. (2021)
	Nucleic acid-lipid particles	–	PEG coating	DOX, siCD47	CD47-SIRPα, CRT-LRP-1α	CT-26-tumor-bearing mice CRT-LRP-1	i.v.	Inhibited tumor growth and prolonged the survival	Abdel-Bar et al. (2021)
Combined with EGFR blockade therapy	EVs	–	–	anti-EGFR/CD47 mAb	CD47-SIRPα, EGFR	4T1 tumor- bearing mice/TNBC patient-derived xenograft mice	i.v.	Suppressed the tumor growth with minimal side effects	Parsa et al. (2007)
Combined with PD-1 blockade therapy	Fusion-CVs fused by SIRPa-CVs and PD-1-CVs	–	–	–	CD47-SIRPα, PD-1-PD-L1	post-surgical 4T1 tumor-bearing mice, B16F10 tumor-bearing mice	i.v.	Inhibited tumor recurrence, promoted overall survival rates by controlling post-surgery recurrence and metastasis	Meng et al. (2021)
	aPD1@aCD47 protein complexes	ROS-responsive	–	–	CD47-SIRPα, PD-1-PD-L1	B16F10 tumor-bearing mice,	i.t.	Activated systemic immune responses to inhibit potential tumor growth and metastasis	Chen et al. (2019c)
	Liposomes	–	PEG coating, aptamer EpCAM conjugation	Si-CD47, si-PD-L1	CD47-SIRPα, PD-1-PD-L1	4T1 tumor-bearing mice/4T1 lung metastatic bearing mice	subcutaneous/i.v.	Inhibited the growth of solid tumors in subcutaneous and reduced lung metastasis in lung metastasis model.	Lian et al. (2019)
	Human serum albumin	pH-responsive	PEG coating, aCD47 conjugation	Dabrafenib, aPD-1	CD47-SIRPα, PD-1-PD-L1, BRAF V600E mutation	B16F10 tumor-bearing mice	i.v.	Suppressed the tumor development with good safety and active targeting	Pham et al. (2021)
	ZIF-8-based nanoparticles	pH-responsive	–	AUNP-12, PQ912	CD47-SIRPα, PD-1-PD-L1,	4T1-tumor-bearing mice	intraperitoneal (i.p.)	Suppressed tumor growth	Zhao et al. (2021)
Combined with PTT	BP-based nanosheets	NIR-responsive	PEG coating	aCD47	CD47-SIRPα	A20 tumor- and metastatic-bearing mice	i.t.	Inhibited primary and metastatic tumor growth	Xie et al. (2020)
	Bismuth selenide nanoparticles	NIR-responsive	PEG coating, aCD47 conjugation	aCD47	CD47-SIRPα	4T1-tumor-bearing mice	i.v.	Resulted in tumor eradication	Guo et al. (2019)
	Silica-core gold nanoshells	NIR-responsive	PEG coating	CD47 mAb	CD47-SIRP	ID8-, TOV21G- and SKOV-3-tumor bearing mice	i.p.	Suppressed tumor growth with less irradiation and a reduced amount of gold nanoshells	Wu et al. (2015)
	Graphene oxide	NIR-responsive	COS grafting, aCD47 conjugation	dacarbazine	CD47-SIRPα mitochondrial apoptosis pathwa	B16F10 cells	co-incubation	Killed the tumor cells	Zhan et al. (2021)

TAMs, tumor associated macrophages; PEG, polyethylene glycol; PTX, paclitaxel; aCD47, anti-CD47, antibody; DBCO, dibenzocyclooctynes; NVs, nanovesicles; plt, platelet; MNPs, magnetic nanoparticles, gCM: genetically engineered cell-membrane; NIR, near infrared radiation; ZIF-8, zinc 2-methylimidazole-8; siIDO-1, small interfering RNA(siRNA) knocking down IDO-1; GE, gemcitabine; CVs, cellular vesicles; ROS, reactive oxygen species; aPD-1, anti PD-1, antibody; EpCAM, epithelial cell adhesion molecule; siCD47, siRNA, knocking down CD47; CRT, calreticulin; LRP-1, low-density lipoprotein receptorrelated protein 1; DOX, doxorubicin; PTT, photothermal therapy.

**FIGURE 3:**
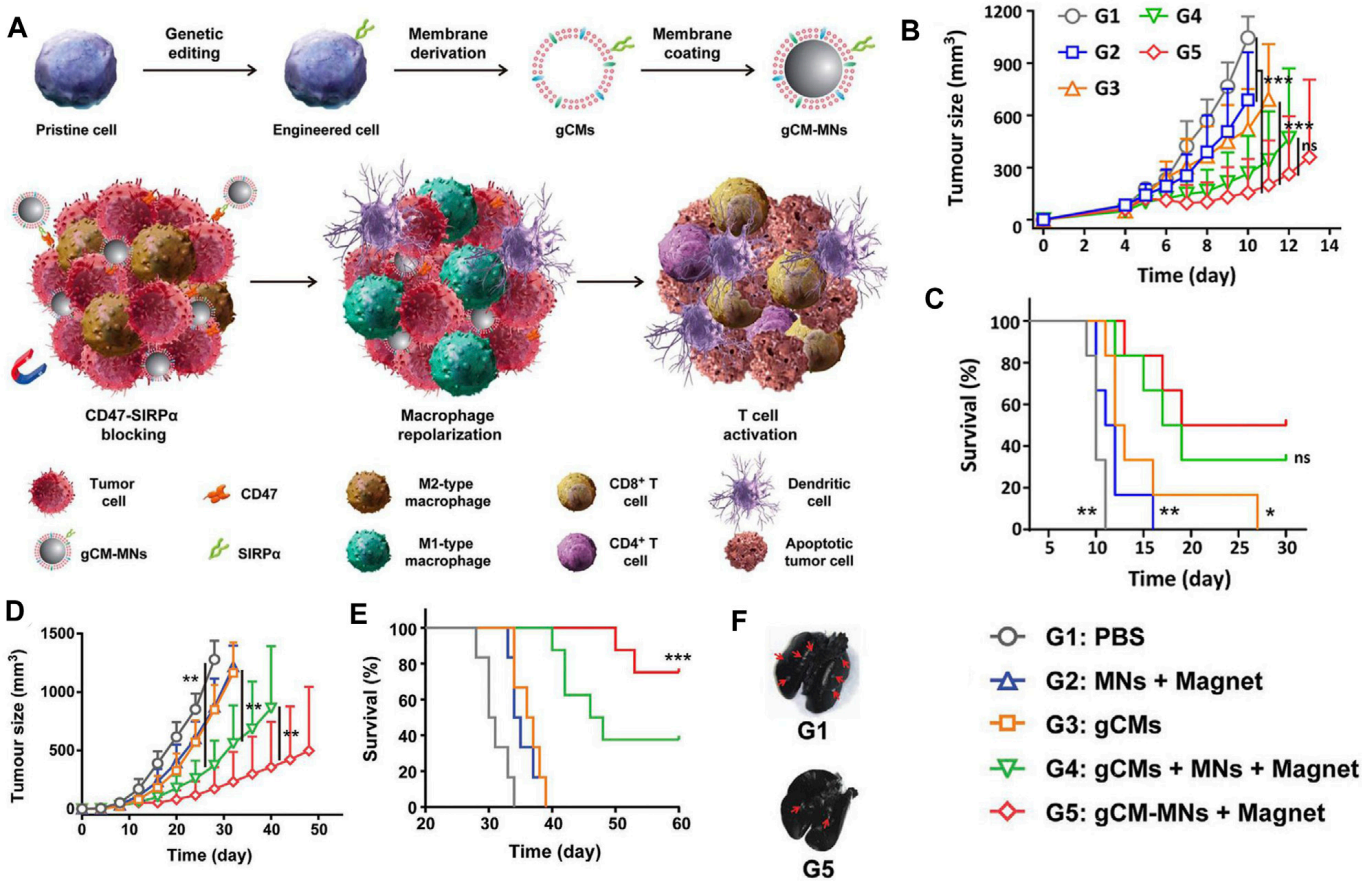
**(A)** Scheme of genetically edited cell-membrane-coated magnetic nanoparticles (gCM-MNs) elicits potent macrophage immune responses for cancer immunotherapy. **(B,C)** gCM-MNs inhibit B16F10 tumor growth. **(B)** Tumor growth kinetics after indicated treatments. **(C)** Survival curves for different treatment groups. **(D–F)** gCM-MNs suppress 4T1 tumor growth and lung metastasis. **(D)** Average tumor growth kinetics after indicated treatments. **(E)** Survival curves for different treatment groups. **(F)** Ink-stained lung photographs for different treatment groups. The red arrowheads indicate tumor foci in the lung. Adapted with permission [59]. Copyright 2020. Wiley.

Recent advances in tumor immunology suggest that the antitumor effect of blocking the CD47-SiRPα signaling pathway may be discounted by the immunosuppressive tumor microenvironment (TME) (Noy and Pollard, 2014; Chen et al., 2019b). In particular, colony-stimulating factors, secreted by tumor cells, are abundant in the TME, polarizing TAMs to the tumorigenic M2 phenotype (Noy and Pollard, 2014; Chen et al., 2019b). M2 TAMs can recruit regulatory T cells (Tregs) and secrete proinflammatory cytokines, all of which impair the activation of CD47 blockers against tumor T-cell immunity (Mantovani et al., 2017; Kulkarni et al., 2018; Feng et al., 2019). In this context, blocking the CD47-SIRPα signaling axis while polarizing tumorigenic M2- to anti-tumor M1-phenotype TAMs can improve the antitumor effect of CD47 immune checkpoint inhibitors (Ramesh et al., 2019; Rao et al., 2020a; Rao et al., 2020b; Chen et al., 2020; Nie et al., 2020; Chen et al., 2021; Huang et al., 2021). For example, Rao et al. have developed a genetically engineered cell-membrane-coated magnetic nanovehicle (gCM-MNs). The gCM shell genetically overexpressing SIRPα variants with prominent affinity and efficiently inhibits the CD47-SIRPα signaling axis, which can also protect the MN core from macrophage phagocytosis. The MN core promotes M2 TAM repolarization, synergistically triggering potent macrophage immune responses. Moreover, the MN core delivers the gCMs into tumor tissues under magnetic navigation, effectively promoting their tumor accumulation. In melanoma and triple-negative breast cancer models, gCM-MNs remarkably extended overall survival by inhibiting local tumor growth and distant metastasis (Rao et al., 2020a; Figure 3).

Many studies have investigated the efficacy of chemotherapy as an adjuvant to immunotherapy, suggesting that the most significant potential mechanism of such adjunct is immunogenic cell death (ICD) (Zitvogel et al., 2008; RA and BW, 2005; Hou et al., 2013). ICD has a number of clearly defined physiological characteristics, including cell surface CRT expression, release of DAMPs such as adenosine triphosphate and heat shock proteins, and release of high mobility group box 1 (Liu et al., 2016). Among these factors, the surface expression of CRT is considered to be the single most important element of ICD (Abdel-Bar et al., 2021). As previously mentioned, the effect of CRT exposure, serving as an “eat me” signal, is considered to be counterbalanced and potentially dampened by CD47 expression (Chao et al., 2010a). Moreover, the upregulated expression of CD47 on the surface of tumor cells makes it an active targeting site for tumor cells, facilitating nonspecific distributed ICD inducing drugs to target tumor tissues and reduce systemic toxicity (Tang et al., 2021). Therefore, codelivery, which simultaneously removes an inhibitory signal and introduces an activating signal, can produce an enhanced antitumor effect (Abdel-Bar et al., 2021; Lee et al., 2021). For example, Abdel-Bar et al. reported a stable nucleic acid-lipid particle (SNALP) formulation with the simultaneous delivery of an ICD inducing drug (Dox) with small interfering RNA (siRNA) knocking down CD47 (siCD47) for synergistic enhancement of ICD. In a CT-26-tumor-bearing mouse model, SNALPs synergistically inhibited tumor growth and prolonged the survival (Abdel-Bar et al., 2021).

EGFR is overexpressed in various solid tumors, such as breast, renal, colon, head and neck cancer (Normanno et al., 2001; Gazdar, 2009; Yu et al., 2013) Hence, EGFR targeting strategy is a promising way for antitumor treatment (Mendelsohn and Baselga, 2003; Yu et al., 2013; Sabbah et al., 2020). CD47 is also overexpressed on the surface of multiple tumor cells. Therefore, dual targeting to EGFR and CD47 strategy can efficiently target and inhibit tumor growth. For example, Si et al. constructed anti-EGFR/CD47 mAb marked EV which showed a high anti-TNBC efficacy with negligible toxicity in both 4T1 tumor-bearing mouse models and TNBC patient-derived xenograft models (Si et al., 2022).

Programmed cell death-ligand 1 (PD-L1) blockade therapy has achieved exciting success in the clinic (Pardoll, 2012). PD-L1, which is highly expressed in many tumor cells, sends a “don’t find me” signal to the adaptive immune system, inhibiting T-cell activation by engaging the PD-1 receptor (Parsa et al., 2007). CD47 sends a “don’t eat me” signal to the innate immune system, inhibiting the phagocytosis of macrophages by engaging SIRPα (Hayat et al., 2020; Logtenberg et al., 2020; Zhang et al., 2020). Hence, dual-blockade of PD-L1 and CD47 can activate potent antitumor effects via both innate and adaptive immune responses (Chen et al., 2019c; Lian et al., 2019; Meng et al., 2021; Pham et al., 2021; Zhao et al., 2021). For example, Meng et al. designed genetically programmable fusion cellular vesicles (Fus-CVs), which were fused by SIRPα variants and PD-1 variants. This bispecific targeting design improves the targeting of tumor cells while reducing the adverse off-target effect on normal cells. In malignant melanoma and mammary carcinoma models, Fus-CVs synergistically suppressed postsurgery tumor recurrence and metastasis, thereby improving overall survival (Meng et al., 2021).

PTT is a promising cancer treatment modality. PTT-induced hyperthermia can be controlled through the local use of photosensitizers and minimally invasive near-infrared (NIR) radiation to reduce damage to untargeted tissues (Sica et al., 2006; Chu and Dupuy, 2014; Chen et al., 2015). Recently, increasing studies have demonstrated that hyperthermia can induce dying tumor cells to release massive amounts of cytokines, such as IL-1β and TNF-α, promoting the immune responses of macrophages, NK cells and T lymphocytes. Yet, it is difficult to completely eradicate large tumors with conventional PTT due to residual tumor mass at the treatment margins (Mantovani et al., 2006; Chu and Dupuy, 2014; Shim et al., 2017). Therefore, researchers combined CD47 blockers with PTT to synergistically enhance the antitumor effect (Wu et al., 2015; Guo et al., 2019; Xie et al., 2020; Zhan et al., 2021). For example, Guo et al. reported bismuth selenide nanoparticles conjugated with anti-CD47 antibody and coated with PEG (Ab-PEG-Bi2Se3). In the 4T1 tumor-bearing model, Ab-PEG-Bi2Se3 plus PTT synergistically eradicate the tumor (Guo et al., 2019).

### Utilizing the “Don’t Eat Me” Signal of the CD47-SIRPα Interaction

**FIGURE 4:**
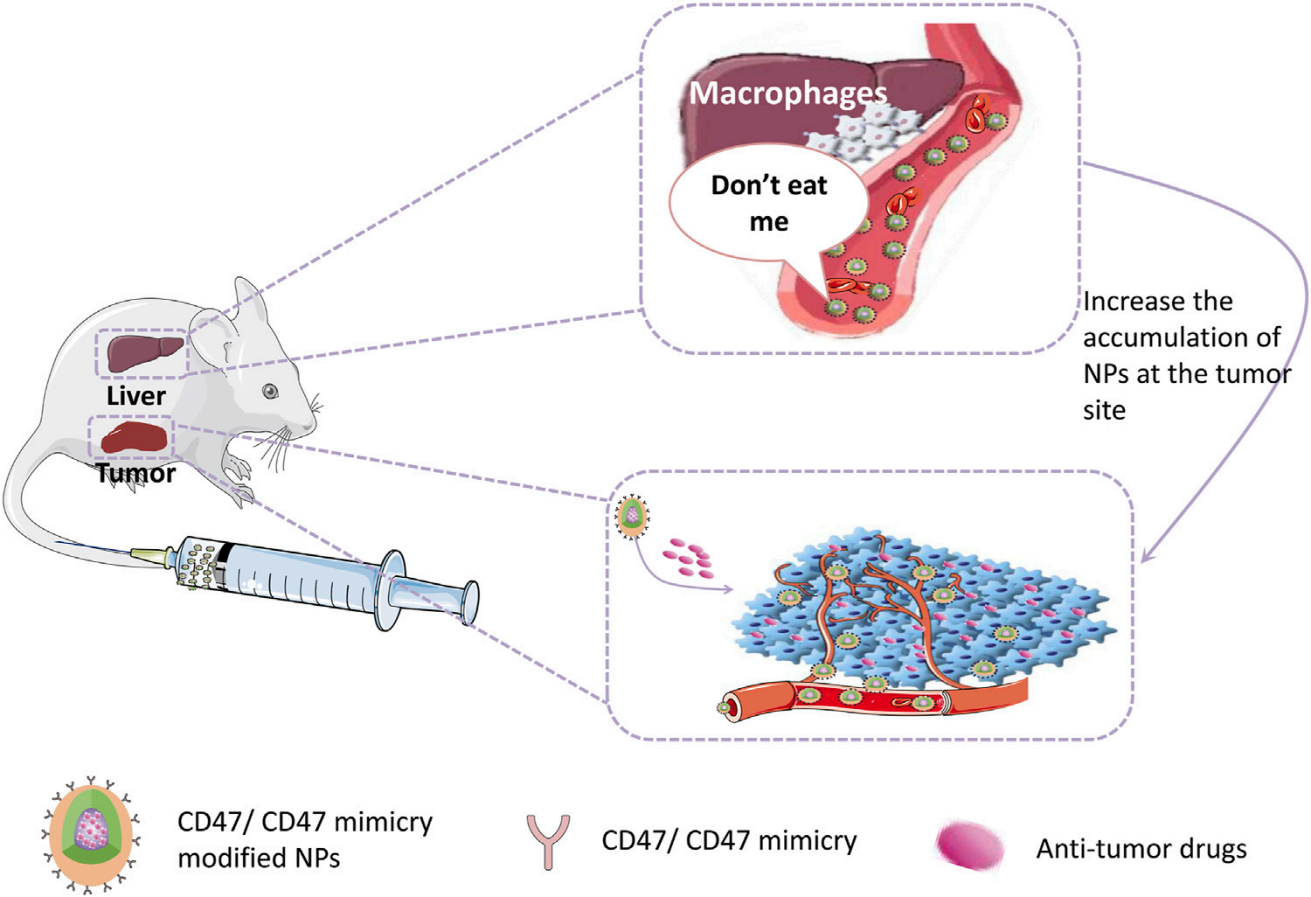
CD47/CD47mimicry modified NPs can evade the phagocytic by reticuloendothelial system which increases their half-life in circulation and the accumulation at the tumor site, hence enhancing the therapeutic effect.

**FIGURE 5:**
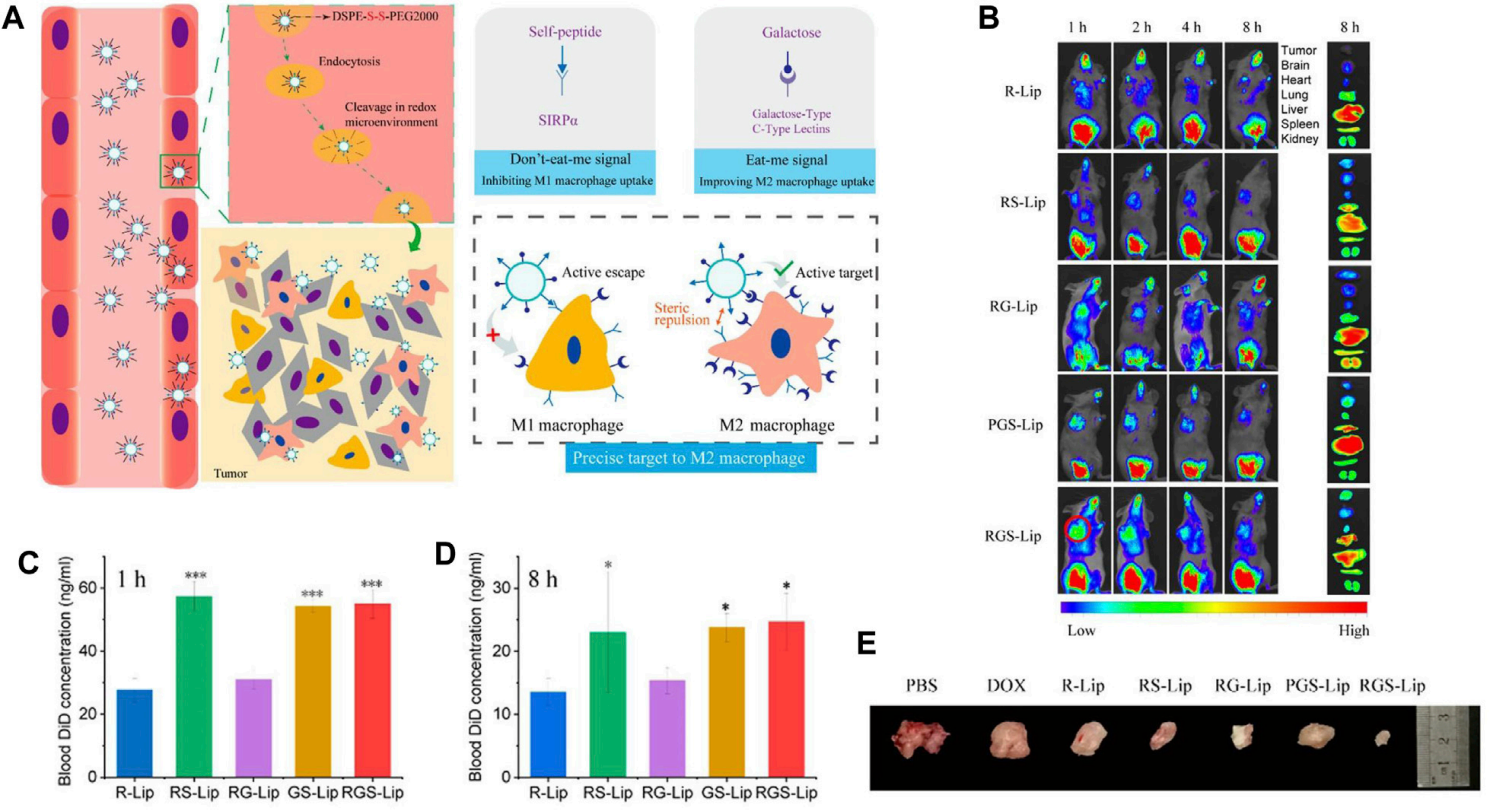
**(A)** Schematic diagram depicting precise delivery of nanomedicine to M2 macrophages. **(B–D)** RGS-Lip prolongs the circulation time and increases the accumulation of liposomes in the tumor. **(B)** *In vivo* and *ex vivo* fluorescence images of liposomes injected into mice. Blood DiD concentration at **(C)** 1 h and **(D)** 8 h. **(E)** Photographs of A20 subcutaneous tumors at the end of treatment. Adapted with permission [98]. Copyright 2020. American Chemical Society.

Reducing the capture of NPs by reticuloendothelial organs (such as liver, spleen, and lung) and extending their circulation time in the blood to accumulate more NPs in tumor tissues have been challenges. Currently, there are many approaches to prolong the half-life of NPs in blood, such as PEG surface modification and bionic membrane coating techniques described above. However, these approaches still have some disadvantages and limitations. Therefore, more suitable alternatives are urgently needed. The CD47 protein, as a “self” marker, can evade phagocytosis by the CD47-SIRPα interaction (Logtenberg et al., 2020). With regard to the pivotal role of CD47 in the regulation of immune responses, the present paper outlines emerging methods for the production of bioinert biomaterials and NPs using CD47 (Gheibi Hayat et al., 2019; Figure 4) Examples of stealth functionalization by CD47 mimicry utilized for antitumor nanomedicine are listed in Table 4 (Kamerkar et al., 2017; Shim et al., 2017; Jiang et al., 2018; Song et al., 2019; Wang et al., 2019; Belhadj et al., 2020; Cheng et al., 2021; Tang et al., 2021; Xie et al., 2021; Zhang et al., 2021). For instance, Tang et al. designed a precise delivery nanomedicine to M2 macrophages by combining “eat me/don’t eat me” signals and verified its role in antitumor therapy in an A20 subcutaneous tumor mouse model. In this delivery system, CD47-derived self-peptide ligand and galactose ligand were introduced on liposomes to reduce the phagocytosis of M1 macrophages and enhance the uptake of M2 macrophages, respectively. Cleavable phospholipid-PEG covering on the surface of liposomes can be removed by the redox microenvironment upon transcytosis through the tumor endothelium and re-expose the self-peptide and galactose. Therefore, this nanocarrier can precisely target M2-type TAMs. In addition, DOX loaded into liposomes further enhances its antitumor effect (Tang et al., 2021; Figure 5)

**TABLE 4:** Examples of nanoparticle-based combination therapy utilized activating CD47-SIRPα signal axis.

Platform type	Responsive release modality	Modification modality	Therapeutic drug	Targets	Tumor model	Administration route	Results	Ref.
Liposome	–	Synergetic-conjugation of aER and CD47 derived SP with PEG	aER, CD47 derived SP, DOX	ER, CD47-SIRPα	MCF-7 tumor- bearing mice	intravenous (i.v.)	Enhanced therapeutic effect of drug delivery via tumor targeting ER and immune clearance-blocking , improved tumor imaging and inhibit tumor growth *via* DOX	Wang et al. (2019)
Hybrid nanovesicle	Thermo-sensitive	Over-expression of CD47 by gene-engineering	ICG and R837	TLR7,CD47-SIRPα	CT26 tumor- bearing mice	i.v	Enhanced therapeutic effect of drug delivery *via* immune clearance-blocking, completely suppressed tumor growth.	Cheng et al. (2021)
Exosomes	–	–	siRNA and shRNA	Oncogenic Kras, CD47-SIRPα	Panc-1 tumor bearing mice	intraperitoneal (i.p.)	Enhanced therapeutic effect of drug delivery *via* immune clearance-blocking, suppressed tumor growth	Kamerkar et al. (2017)
Nanosheet	–	CD47 derived SP	–	CD47-SIRPα	SCC7 tumor- bearing mice	i.v.	Reduced the non-specific phagocytosis of nanosheets by macrophages, increased the blood circulation time and nanosheets uptake by tumor cells.	Shim et al. (2017)
EVs; Hybrid vesicles	–	Cationized mannan; c (RGDm7)	DOX, GE	Mannose, CD47-SIRPα, EGFR	A549 tumor- bearing mice	i.v.	Reduced endocytosis of macrophages, increased the blood circulation time and nanosheets uptake by tumor cells, suppressed the tumor growth	Zhao et al. (2021)
Liposomes	Redox responsive	CD47 derived SP, galactose ligand, PEG	DOX	Galactose, CD47-SIRPα	A20 tumor- bearing mice	i.v.	Preferentially reduced M1 macrophage phagocytosis and selectively killed M2 macrophages and tumor cells, synergically enhanced the anti-tumor efficacy	Tang et al. (2021)
Ellipsoidal PLGA	–	CD47-Fc, H-2Kb/TRP2180-188-Ig dimers, anti-CD28 ,PEG	–	CD47-SIRPα	B16F10 tumor-bearing mice	i.v.	Minimized cellular uptake of nano-aAPCs and enhanced their functionality to expand antigen-specific T cells and inhibits tumor growth	Song et al. (2019)
Micelles	pH responsive	CD47 derived SP coating, AP	DOX/SPION	CD47-SIRPα, Y_1_ receptorα	MCF-7 tumor-bearing nude mice	i.v.	Reduced the accumulation of micelles in liver and kidney, enhanced the specific targeting and high retention of SPION or DOX loaded micelles in tumor sites, generating excellent MR signal and therapeutic efficacy with prolonged survival time *in vivo*.	Jiang et al. (2018)
Porous silicon particles	–	CD47 derived SP coating, YIGSR peptide	AS1411, tanespimycin	CD47-SIRPα, β1-integrine	HOS-MNNG tumor-bearing nude mice	i.v.	Reduced the accumulation of NPs in the liver, improved the tumor targeting and suppressed the tumor growth	Zhang et al. (2021)
CD47-positively tumor-derived exosomes	–	–	DOX	CD47-SIRPα	MDA-MB-231-bearing nude mice	i.v.	Prevented breast cancer metastasis to the lungs	Xie et al. (2021)

aER, anti-ER antibody; PEG, Polyethylene glycol; SP, self-peptide; DOX, doxorubicin; siRNA, small interfering RNA; shRNA, short hairpin RNA; EVs, extracellulr vesicles; GE, gefitinib; PLGA, poly (lactic-co-glycolic acid); AP, (Asn6, Pro34)-NPY; SPION, super-paramagnetic iron oxide nanoparticle.

## Conclusion and Limitations

CD47 is an inhibitory immune checkpoint that is highly expressed on tumor cells, binding with SIRPα on myeloid cells and thereby releasing a “don’t eat me” signal, inhibiting phagocytosis. On one hand, blocking the CD47-SIRPα signaling axis can activate macrophage phagocytosis of tumor cells and enhance the antigen presenting function of DC, subsequently bridging innate immune responses with the adaptive immune responses. Therefore, tumor immunotherapy focusing on the CD47-SIRPα axis has recently garnished significant attention. However, due to nonspecific targeting, systemic administration of CD47-SIRPα blockades can cause severe side effects, which provides the development of nanomedicine a great opportunity. On the other hand, researchers make use of this negative regulatory effect of the CD47-SIRPα axis to decorate the NPs with a stealthy function, markedly increasing both circulation time and drug uptake by tumor cells. Due to the high plasticity and selectivity of nanomaterials, they can be used as therapeutic agents (such as CD47-rich vesicles) and drug delivery vehicles of any site of the CD47-SiRPα signaling axis. It offers great convenience for the realization of targeted therapy, combination therapy and the improvement of antitumor effect.

Although anticancer nanomedicine focusing on this signaling axis has extensive prospects, there are still many challenges to be overcome to realize full practical applications. For example, understanding of the CD47-SiRPα signal axis is not thorough enough, such as how to properly regulate the intensity of this signal axis in the spatiotemporal category. In addition, the synthesis of ideal NPs is complex and difficult. As mentioned earlier, small changes in any part of the NP manufacturing process can lead to large changes in the performance of nanomedicines. This sensitivity requires the knowledge of nanomaterials and rigor in the fabrication process. The harm of nanomaterials cannot be neglected. Last but not least, regarding combination therapy, control of the drug loading ratio and the spatiotemporal order of drug release are problems to be solved.

Nevertheless, these challenges can also be opportunities. With the deepening of researchers’ knowledge of the CD47-SiRPα pathway and tumor nanomedical science, the perfect combination of increasingly mature nanotechnology and body pathology and physiology in the future will achieve better clinical transformation.

## Data Availability

The original contributions presented in the study are included in the article/Supplementary Material, further inquiries can be directed to the corresponding author.
